# The Influence of Different Recycling Scenarios on the Mechanical Design of an LED Weatherproof Light Fitting

**DOI:** 10.3390/ma7085769

**Published:** 2014-08-11

**Authors:** Víctor Camañes, Daniel Elduque, Carlos Javierre, Ángel Fernández

**Affiliations:** 1 Zalux, Centro Empresarial Miralbueno, 50012 Zaragoza, Spain; E-Mail: victor.camanes@gmail.com; 2 i+, Department of Mechanical Engineering, University of Zaragoza, C/María de Luna, 3, 50018 Zaragoza, Spain; E-Mail: daniel.elduque@gmail.com; 3 AITIIP Technology Center (Centro Tecnológico en Inyección de Plásticos), Polígono Industrial Empresarium, C/Romero 12, 50720 Zaragoza, Spain; E-Mail: angel.fernandez@aitiip.com

**Keywords:** recycling, life cycle assessment (LCA), light fitting

## Abstract

This paper analyzes the high relevance of material selection for the sustainable development of an LED weatherproof light fitting. The research reveals how this choice modifies current and future end of life scenarios and can reduce the overall environmental impact. This life cycle assessment has been carried out with Ecotool, a software program especially developed for designers to assess the environmental performance of their designs at the same time that they are working on them. Results show that special attention can be put on the recycling and reusing of the product from the initial stages of development.

## 1. Introduction

Environmental conscience has been growing gradually in recent decades. In the last few years, several laws have been developed in the European Union in order to reduce the environmental impact of consumer products. These laws are devoted to reduce the use of hazardous substances [1], control chemicals [2], enhance the recycling (WEEE, waste of electrical and electronic equipment) [3] and introduce ecodesign in energy-using and energy-related products [4,5].

This paper analyzes the high relevance of material selection and how this choice modifies end of life scenarios, being possible to reduce the environmental impact at the end of life of the product. The paper is focused on analyzing the environmental impact of the product, changing the end of life scenarios in accordance with different business models, analyzing the recycling impact depending on the selected materials by means of a simplified life cycle assessment (LCA) analysis. This analysis has been performed over an LED weatherproof luminaire produced by Zalux S.A. (Alhama de Aragon, Spain). This Spanish company produces more than four million luminaires per year.

The industrial luminaire is a product massively produced around the world. This product is used for different purposes, like lighting in garages, factories and several places where the product is going to be used during the years, or also, it can be used as provisional lighting systems, like construction lighting. A lighting system built with this kind of luminaire can use hundreds of them, using a large amount of material and energy consumption; so, due to the large quantity of luminaries used around the world, the optimization of the environmental impact of this product is important, reducing the environmental impact at all of the different phases of the life cycle, this being a benefit for society.

**Figure 1:**
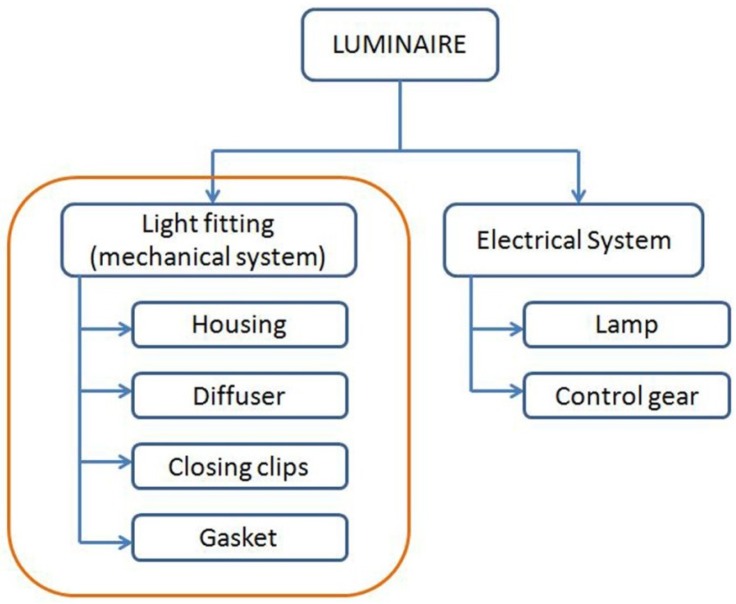
Product structure.

These weatherproof luminaires are composed of two main parts: the electrical system, composed of the lamp and the control gear, and the light fitting, which is the mechanical system that protects the electrical one. The main parts of a light fitting are the housing, where the electrical system is fixed, the diffuser, which closes the luminaire and diffuses the light, closing clips and a gasket, which ensures the watertightness. Figure 1 shows the product structure, with the parts we have focused our efforts on in this paper mentioned.

Traditionally, these weatherproof light fittings were produced with plastics and designed as a simple box, where the lighting system was introduced, but nowadays, due to the LED revolution, the technology of these light fittings is getting more complicated. An LED generates between a 60%–70% heat from the power it consumes. As far as semiconductor technology is very temperature sensitive, this heat reduces its performance and its lifetime, so heat dissipation has become the key to success when developing luminaires with LED technology [6,7]. New materials are being used, like aluminum for the housings to improve heat dissipation for the LED lamp, or polycarbonates (PC’s) and polymethyl methacrylates (PMMA’s) for the light diffuser, to increase the efficiency of the luminaire by means of a good light transmittance. The evolution of LEDs is making it possible to use polymers also for the housing, as far as they are being thermally improved, so that there are different possibilities when choosing a material. Furthermore, to protect the LED from inappropriate handling, these light fittings are being designed to be maintenance free, that is, the light fitting cannot be opened; so, when it reaches its end of life, the whole luminaire is discarded without repairing it.

There is potential for improvement in order to reduce the environmental impact of these luminaires. The biggest efforts are usually focused on the efficiency of the lighting system, reducing the environmental impact during the use phase of the product, where the main environmental impact is produced [8,9,10,11]. In spite of that, the other phases of the life cycle should not be overlooked, as improvements in the mechanical components can also be used to reduce the environmental impact of LED luminaires. Apart from changing consumer behavior, the electricity mix or improving LED efficiency to reduce environmental impact at the use phase, the other way to reduce the environmental impact is by applying design actions to the product through its whole life-cycle.

There is room for improvements on designing the light fittings to reduce the environmental impact of its production processes, reducing the transport impact by means of weight reductions and also very interesting ways of reducing the environmental impact, paying attention to the end of life phase. The selection of the material is a key decision, as far as it is going to affect the weight of the part, the functional specifications, costs and environmental impact. Depending on the selected material, the end of life actions that can be applied to the product could be different.

When the light fitting reaches the end of life, depending on the selected materials, different actions can be applied. There are materials that are easily recycled, like aluminum, but others, like PC, could be recycled, but are not usually recycled. On the other hand, materials, such as sheet molding compound (SMC), cannot be recycled and are sent to incineration or land filling. An adequate material selection will affect the environmental impact, particularly at the end of life of the product if it is recycled.

The recycling of plastics is a complex process, as the properties can change from the ones of the virgin material [12]. This means that to improve recycling efficiency, designers have to understand the recycling processes, avoiding contaminants [13,14]. For example, polyethylene terephthalate (PET) recycling is hugely extended, using super-clean recycling that decontaminates post-consumer contaminants, making it food contact approved [15]. The recycling of other plastic is not as extended, although it has been broadly studied also for new materials, like bioplastics [16]. In other cases, instead of mechanical recycling, chemical recycling has also been applied [17,18,19]. Plastic waste management systems have been widely studied [20,21,22,23]. Furthermore, closed-loop systems have been analyzed for several materials, including plastics containing flame retardants [24].

This closed-loop system is a business strategy where the product is recovered by the company when it reaches its end of life, to be replaced by a new one, and the old product is processed by the company. This system brings to the company the possibility of recycling the materials on its own, to be reused in their processes, this being a possibility to save money on raw materials, reducing the environmental impact and giving the customer an additional service.

Life cycle assessment (LCA) is a methodology that allows researchers to calculate the environmental burden created by a product. It is very adaptable for used in different products and services. It has been used to assess products, such as wind turbines [25], electronic boards [26], nanomaterials [27], food packaging [28] or concrete [29]. The recycling of multiple polymer materials has been analyzed using LCA [30]: Polyvinyl chloride (PVC) [31], PET [32], PMMA [33] and even mixed plastic waste [34].

Life cycle assessment has also been used to compare virgin and recycled polymers, finding clear environmental advantages [35,36]. A comparison for TV sets between mechanical recycling and energy recovery was performed, finding that the number of different plastics used in a product should be reduced [37]. Closed-loop recycling was the better environmental option analyzed by [38]. Although downcycling, recycling with a loss of properties, is currently well known, there are not many examples of product upcycling [39,40,41].

This paper presents the first analysis of the differences of the environmental impact of a light fitting when changing the materials, paying special attention to the end of life, analyzing how the environmental impact is modified by changing the materials. The selection of materials and the end of life scenario is linked to the business strategy, and a closed-loop system is proposed. Three different end of life scenarios have been analyzed, to see the difference in the environmental impact of the product when analyzed following IEC (International Electrotechnical Commission) TR62635 [42], and also analyzed in accordance with the proposed end of life scenarios in accordance with the closed-loop business strategy.

This environmental impact analysis has been performed by means of the LCA methodology, as the first environmental viability analysis. It has been applied by means of a software program, called Ecotool, developed by the authors. This software is particularly designed to help mechanical designers to perform the environmental assessment of their designs, being applied during the development stage of a product. Several researchers have found that most life cycle assessment tools focus on analyzing existing products, but they are not suitable for designers [43]. This software allows one to easily configure the LCA inventory and to calculate the environmental impact, by means of simple data input windows.

## 2. Life Cycle Assessment Methodology

### 2.1. System Boundaries

From the industrial luminaire, only the light fitting has been taken into account, as far as this part is the one produced in Zalux, and all of the analysis is going to be focused on the material selection process and the end of life of the mechanical parts. The electrical components are not analyzed in this paper. The analyzed parts are the housing, the diffuser, the closing clips and the weatherproof gasket, as shown in Figure 1 and Figure 3.

**Figure 2:**
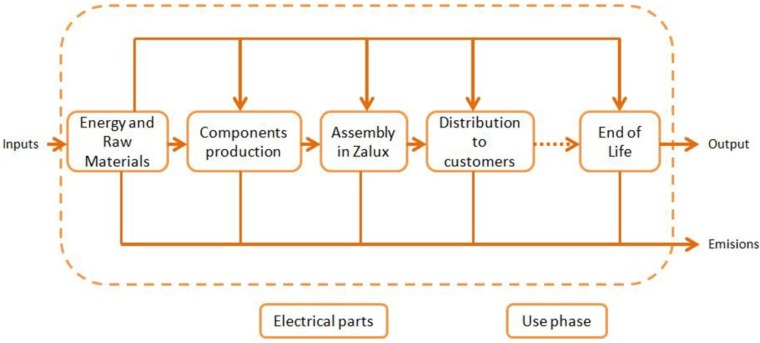
Life cycle system boundaries

Performing this LCA, the whole life cycle has been considered, leaving out of the boundaries the use phase, affected by the electrical gear, not included within the components of a light fitting (Figure 2). Furthermore, outside the limits of the system fall maintenance operations, as LED luminaires are expected to be maintenance-free. These components are produced in Spain and assembled in the Zalux factory (Alhama de Aragon, Spain). The temporal scope is limited to the year 2013, as data fir this year were readily available.

### 2.2. Functional Unit

**Figure 3:**
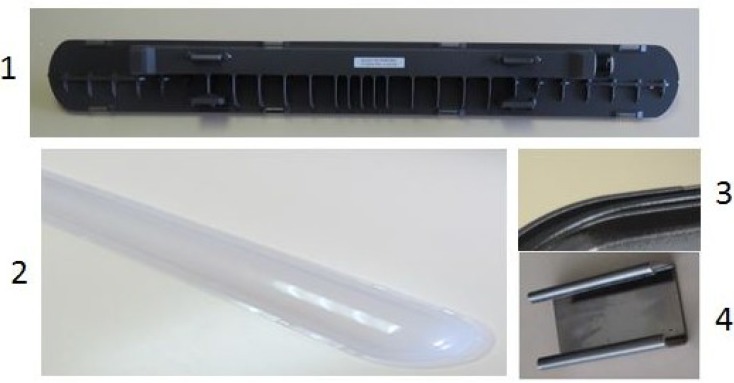
Parts of the light fitting: (**1**) housing; (**2**) light diffuser; (**3**) gasket; (**4**) closing clips.

The functional unit has been chosen to be an industrial weatherproof light fitting composed of a housing, a light diffuser, 8 clips for the light fitting closing and a weatherproof gasket; placed in an average European consumer product. The following picture (Figure 3) shows the parts analyzed in this paper.

### 2.3. Inventory Data

This paper has been carried out in collaboration with Zalux S.A., the company that develops, manufactures and sells these weatherproof light fittings. Most information has been provided from internal data from the company and their suppliers. The use phase has not been taken into account, since the environmental impact of this phase is related only to the electronic components, which are not analyzed in this paper. We have focused on the mechanical parts, as these are the only ones designed and manufactured by the company and, consequently, the ones that could be improved after performing the LCA.

All of the different parts of the light fitting have been weighed. Different materials have been considered for each part to analyze the influence of the material selection over the environmental impact. Each material has different properties, but all of the proposed materials are suitable to be used in a light fitting, as far as the company already uses these materials in different light fittings. For those materials that are not currently used to produce the analyzed light fitting, the weight has been calculated from the volume of the parts obtained from 3D models and the density of the material.

The transport of the product from the factory to the consumer has been considered in all of the scenarios. For those scenarios with the closed-loop system, this transport will be increased according to the percentage of light fittings taken back to the factory.

Different end of life scenarios have been studied; as Europe is the area where the biggest percentage of sales is concentrated, it is supposed that all of the light fittings are going to be processed in WEEE plants.

Life cycle inventory was developed using EcoInvent v3.0. This Swiss database is currently used worldwide. The assignation between inventory data and database was performed following the guidelines provided by [44].

### 2.4. Assessment Methods

The life cycle assessment has been calculated using CML (Institute of Environmental Sciences) Leiden global warming as the midpoint category [45] and ReCiPe (H/A) as the endpoint category [46]. The ReCiPe methodology has been chosen, because it combines scientific soundness with an easy understanding of the results, thanks to the combination of the CML2001 and EcoIndicator99 methods, calculating an endpoint value out of 18 different environmental categories. The global warming category has also been chosen, because of its social relevance and as the use of this metric in companies is currently encouraged by Spanish laws [47].

### 2.5. The Software Ecotool

The LCA has been performed with this software, developed by the research team. This software uses the database, EcoInvent v3.0, to obtain the environmental burdens necessary for the environmental impact calculation.

The software has been developed as an environmental assessment tool to be used especially by mechanical engineers/designers. The databases are customized for the product that is going to be analyzed, so that the environmental assessment process is very easy to be performed, as long as the designer does not need to work with huge databases with thousands of different datasets, which helps the designer to save time when performing an LCA while developing a new product.

The software requires the following inputs: weight, material, end of life configuration, processes and transports. All of this information is easy to collect by the designer when developing a product.

## 3. Life Cycle Inventory

This section presents all of the information used to perform the environmental impact calculation. For each part, the materials, the production processes and the end of life scenario has been collected. The transportation to customers has been taken into account, as far as the total weight of the light fitting has a direct effect on the impact of this transport.

All of this information has been structured as follows.

- 3.1. Components manufacturing
- 3.2. Distribution to consumers
- 3.3. End of life data
  ο 3.3.1. Scenario 1
  ο 3.3.2. Scenario 2
  ο 3.3.3. Scenario 3

### 3.1. Components Manufacturing

In this section, all of the information related to the materials and the production processes of the different parts is presented. These parts are shown in Figure 3. Optional components have not been included in the analyzed light fitting. The following table (Table 1) shows the different materials that can be used for each part, with their different weights, and shows also the production process of each part according to the material.

**Table 1:** Alternative part materials. PC, polycarbonate; SMC, sheet molding compound.

Part	Material	Weight (g/part)	Number of Parts	Process
**Housing**	PC	430	1	Injection molding
	SMC	550	1	Thermal Compression
	Aluminum	1400	1	Injection molding
**Diffuser**	PC	310	1	Injection molding
	PMMA	310	1	Injection molding
	Styrene-acrylonitrile (SAN)	265	1	Injection molding
**Clips**	Polyamide 6 glass-filled (PA6 GF10)	2.5	8	Injection molding
	Stainless steel	3.8	8	Press
**Gasket**	Polyurethane (PU) flexible foam	18	1	Heat curing

**Figure 4:**
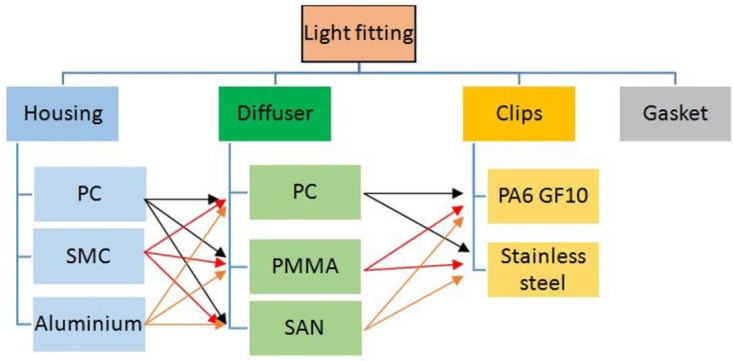
Design combinations.

There are 18 possible combinations (Figure 4). Depending on the material selection, 18 different light fittings can be configured, so 18 LCA models could be calculated.

The input data for the environmental impact calculation has been taken from the EcoInvent 3 database. The following tables (Table 2 and Table 3) show the dataset info for the most relevant material and production process.

**Table 2:** Material datasets.

Material	EcoInvent Dataset
**PC**	Polycarbonate production, RER (Europe)
**SMC**	Glass fiber-reinforced plastic production, polyester resin, hand lay-up, RER
**PMMA**	Polymethyl methacrylate production, beads, RER
**SAN**	Styrene-acrylonitrile copolymer production, RER
**PA6 GF 10**	Nylon 6 production, glass-filled, RER + Nylon 6 production, RER
**Aluminum**	Aluminum production, primary, ingot, GLO (Global)
**Stainless steel**	Steel production, chromium steel 18/8, hot rolled, RER
**PU flexible foam**	Polyurethane production, flexible foam, RER

**Table 3:** Process datasets.

Process	EcoInvent Dataset
**Injection molding**	Injection molding, RER
**Electricity consumption**	Market for electricity, medium voltage, ES (Spain)

### 3.2. Distribution to Consumers

Zalux sells its light fittings worldwide, with the biggest amount of sells concentrated in Europe. With the selling volumes in the different countries, provided by the company, an average distance of 1900 km has been calculated to transport the product from Alhama de Aragon (Spain), where the factory is placed, to the customers. The product is transported by truck, and the Ecoinvent Dataset for this transport is: Transport, freight, lorry > 32 metric ton, EURO4, RER.

### 3.3. End of Life Data

Different end of life scenarios have been analyzed. In the first step, the end of life scenarios established by IEC TR62635 for the different materials have been calculated. Another two scenarios have been performed, thinking about new market strategies and the closed-loop, looking for an improvement on the recycling of the product. Table 4 shows the datasets from EcoInvent for incineration and landfilling.

**Table 4:** End of life datasets.

Material	Incineration	Landfilling
**Stainless Steel**	-	Treatment of scrap steel, inert material landfill (GLO)
**Aluminum**	-	Treatment of waste aluminum, sanitary landfill (GLO)
**SMC**	Treatment of waste plastic, mixture, municipal incineration (GLO)	Treatment of waste plastic, mixture, sanitary landfill (GLO)
**PC**		
**PMMA**		
**SAN**		
**PA GF10**		
**PU**	Treatment of waste polyurethane, municipal incineration (GLO)	Treatment of waste polyurethane, sanitary landfill (GLO)

For those scenarios, EcoInvent guidelines for recycling have been followed. Where plastic recycling is considered, milling, which is a mechanical plastic recycling process currently used by the company, is the recycling method we have considered. This recycling process has a medium voltage electricity consumption of 0.6 kWh/kg, avoiding the production of raw material. Metal recycling has been analyzed using primary and scrap datasets to model the recycling process.

A new part cannot directly be produced entirely with recycled material, but mixing it with raw material, the final properties of the mixture can be suitable to comply with the functional specifications of the product. Percentages around 40%–50% of recycled material could be reached, this being a savings for the company not using only raw material in the production process, and also, this is a new possibility to avoid downcycling when recycling materials [48].

This closed-loop system can reduce the environmental impact of the product, recycling material when the IEC TR62635 sets that it is not recycled, as far as the company can ensure that the material is going to be properly recycled in its production process.

#### 3.3.1. Scenario 1: IEC TR62635

This end of life scenario is established following the IEC TR62635, which sets the different possible end of life for the materials (recycling, incineration or land filling). The following table (Table 5) shows the percentages for each material of their end of life scenario.

#### 3.3.2. Scenario 2: Half Closed-Loop System

In this scenario, a closed-loop system is presented. When the light fitting reaches its end of life, the company supplies the customer a new light fitting, and the installer takes the light fitting back to the factory to recycle the materials, mixed with raw material in the appropriated proportions, to produce new parts. In this scenario, it has been established that half of the light fitting will be treated in a closed looped system, and the other half will have the Scenario 1 end of life, assuming that the company cannot have control over the whole sold light fitting. As SMC and the polyurethane gasket cannot be recycled, due to the properties of the materials, its recycling percentage has not been increased. The following table shows the percentage of material sent to the different end of life scenarios (Table 6).

**Table 5:** End of life Scenario 1.

Part	Material	End of Life Scenario		
		Recycling (%)	Incineration (%)	Land Fill (%)
**Housing**	PC	0	5	95
	SMC	0	5	95
	Aluminum	91	0	9
**Diffuser**	PC	0	5	95
	PMMA	0	5	95
	SAN	0	5	95
**Clips**	PA6 GF10	0	5	95
	Stainless steel	94	0	6
**Gasket**	PU flexible foam	0	5	95

**Table 6:** End of life Scenario 2.

Part	Material	End of Life Scenario		
		Recycling (%)	Incineration (%)	Land Fill (%)
**Housing**	PC	50	5	45
	SMC	0	5	95
	Aluminum	95.5	0	4.5
**Diffuser**	PC	50	5	45
	PMMA	50	5	45
	SAN	50	5	45
**Clips**	PA6 GF10	50	5	45
	Stainless steel	97	0	3
**Gasket**	PU flexible foam	0	5	95

#### 3.3.3. Scenario 3: Whole Closed-Loop System

A complete closed-loop system has been applied in Scenario 3. In this scenario, it is considered that all of the old material is recycled and mixed with raw material in the appropriated proportions to produce new parts. Again, SMC and the gasket are not recycled. The following table (Table 7) shows the end of life percentage for each part together with the material.

**Table 7:** End of life Scenario 3.

Part	Material	End of Life Scenario		
		Recycling (%)	Incineration (%)	Land Filling (%)
**Housing**	PC	100	0	0
	SMC	0	5	95
	Aluminum	100	0	0
**Diffuser**	PC	100	0	0
	PMMA	100	0	0
	SAN	100	0	0
**Clips**	PA6 GF10	100	0	0
	Stainless steel	100	0	0
**Gasket**	PU flexible foam	0	5	95

## 4. Results and Discussion

In this section, the results of each scenario are shown. In order to reduce the number of combinations shown in Figure 4, the housing, the diffuser, the gasket and the clips are going to be analyzed separately.

The material, processes and end of life environmental impact are going to be calculated for each part. Manufacturing is the result of adding the environmental impact of the material and the production process, and the end of life is calculated in accordance with the configured scenarios shown in Section 3.3

Then, the complete light fitting analysis will be performed with the assembly of the best combination for the different parts, adding the transportation to customers, using the weight of the optimum assembly, so that the transport environmental impact will be calculated with the total weight of the light fitting. The calculation procedure is as follows: calculation of the environmental impact of the materials, end of life and processes of each part for the different materials.

(1) Selection of the lowest impact part depending on the material.
(2) Assembly of the light fitting with the lowest impact parts, and calculation of the environmental impact of transport to customer.

Then, we calculate the total impact by adding manufacturing, transport to customers and end of life.

### 4.1. Scenarios Results

#### 4.1.1. Scenario 1: IEC TR62635

Table 8 shows the result of the individual analysis of each part. The parts with a lower impact are green colored.

The total weight of the light fitting is 863.4 g. The environmental impact of the transport to the customer is 18.96 mPt (ReCiPe) and 0.18 kg_eq_CO_2_. Therefore, the total impact of the light fitting is:

Total impact (ReCiPe) = Manufacturing + Transport + End of life = 456.96 mPt

Total impact (kg_eq_CO_2_) = Manufacturing + Transport + End of life = 4.60 kg_eq_CO_2_

**Table 8:** Results for Scenario 1.

Part	Manufacturing (Materials + Production)		End of Life	
	ReCiPe (mPt)	Carbon Footprint (kg_eq_CO_2_)	ReCiPe (mPt)	Carbon Footprint (kg_eq_CO_2_)
Clip plastic	15.4	0.24	0.24	0.8
Clip steel	36.7	0.16	−30.8	−0.08
Diffuser SAN	142	1.38	4.46	0.07
Diffuser PMMA	239	2.55	4.56	0.06
Diffuser PC	237	2.75	4.56	0.06
Gasket	9.80	0.21	0.28	0.01
Housing SMC	267	2.59	8.09	0.11
Housing PC	329	3.82	6.33	0.08
Housing Aluminum	2412	25.3	−1924	−20.7

**Figure 5:**
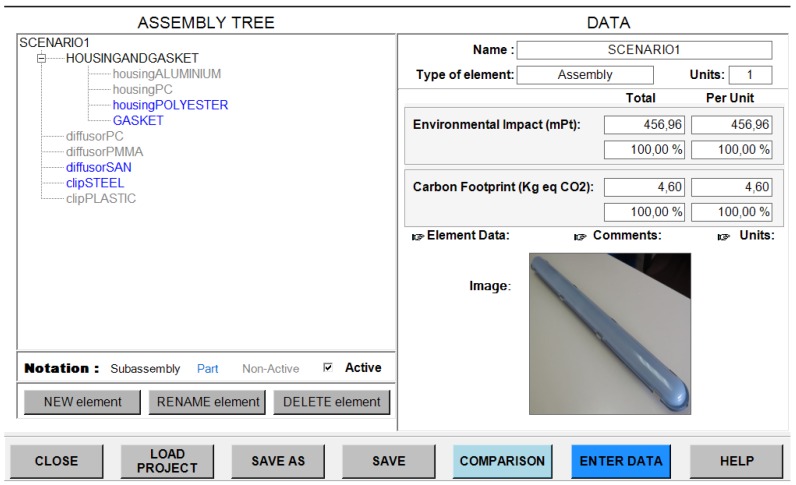
Results for Scenario 1.

The following picture (Figure 5) shows the software used to calculate the environmental impact, where the final design assembly is shown, with the parts colored in blue. The final results for ReCiPe and for carbon footprint are shown in the picture.

#### 4.1.2. Scenario 2: Half Closed-Loop System

As for Scenario 1, the following table (Table 9) shows the individual results for each part with the new end of life scenario, with the lower impacting parts green colored.

For this scenario, the environmental impact of the transport to the customer is multiplied by 1.5, as far as half of the light fittings return to the factory, so that half of the environmental impact of the transport to the customer is added again in returning operation of the old product.

**Table 9:** Results for Scenario 2.

Part	Manufacturing (Materials + Production)		End of Life	
	ReCiPe (mPt)	Carbon Footprint (kg_eq_CO_2_)	ReCiPe (mPt)	Carbon Footprint (kg_eq_CO_2_)
Clip plastic	15.4	0.18	0.29	0.0
Clip steel	36.7	0.16	−31.8	−0.12
Diffuser SAN	142	1.38	−47.8	−0.43
Diffuser PMMA	239	2.55	−92.9	−0.99
Diffuser PC	237	2.75	−92.2	−1.09
Gasket	9.80	0.21	0.28	0.01
Housing SMC	267	2.58	8.09	0.11
Housing PC	329	3.82	−128	−1.51
Housing Aluminum	2412	25.3	−2019	−21.7

The total weight of the light fitting is 743.4 g. The environmental impact of the transport to the customer is 16.3 mPt (ReCiPe) and 0.15 kg_eq_CO_2_, but taking into account the returning to the factory of half of the light fittings, the total transport impact is 24.5 mPt (ReCiPe) and 0.23 kg_eq_CO_2_. Therefore, the total impact of the light fitting is:

Total impact (ReCiPe) = Manufacturing + Transport + End of life = 336 mPt

Total impact (kg_eq_CO_2_) = Manufacturing + Transport + End of life = 3.74 kg_eq_CO_2_

#### 4.1.3. Scenario 3: Whole Closed-Loop System

The analysis of this scenario follows the same steps as Scenario 2, but changing the end of life scenario as shown in Section 3.3. The following table (Table 10) shows the individual results for each part with the new end of life scenario, with the lower impacting parts green colored.

**Table 10:** Results for Scenario 3.

Part	Manufacturing (Materials + Production)		End of Life	
	ReCiPe (mPt)	Carbon Footprint (kg_eq_CO_2_)	ReCiPe (mPt)	Carbon Footprint (kg_eq_CO_2_)
Clip plastic	15.5	0.18	0.3	0
Clip steel	36.7	0.18	−32.8	−0.12
Diffuser SAN	142	1.38	−102	−0.98
Diffuser PMMA	239	2.55	−192	−2.08
Diffuser PC	237	2.75	−191	−2.28
Gasket	9.80	0.21	0.28	0.01
Housing SMC	267	2.58	8.09	0.11
Housing PC	329	3.82	−264	−3.16
Housing Aluminum	2412	25.3	−2114	−22.8

For this scenario, the environmental impact of the transport to customer is multiplied by 2, as far as all the light fittings turn back to the factory, so all the environmental impact of the transport to customer is added again in the turning back operation of the old product.

The total weight of the light fitting is 743.4 g. The environmental impact of the transport to customer is 16.3 mPt (ReCiPe) and 0.15 kg_eq_CO_2_, but taking into account the turning back to the factory of the half of the light fittings, the total transport impact is 32.7 mPt (ReCiPe) and 0.31 kg_eq_CO_2_. So, the total impact of the light fitting is:

Total impact (ReCiPe) = Manufacturing + Transport + End of life = 152 mPt

Total impact (kg_eq_CO_2_) = Manufacturing + Transport + End of life = 1.64 kg_eq_CO_2_

### 4.2. Results Comparison

**Figure 6:**
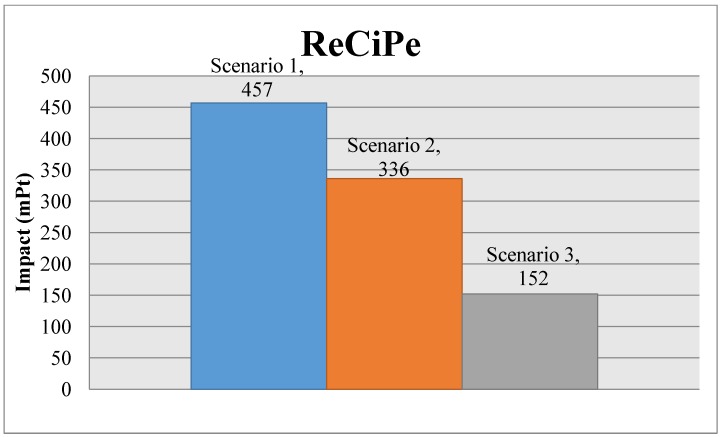
Comparative between scenarios, ReCiPe.

**Figure 7:**
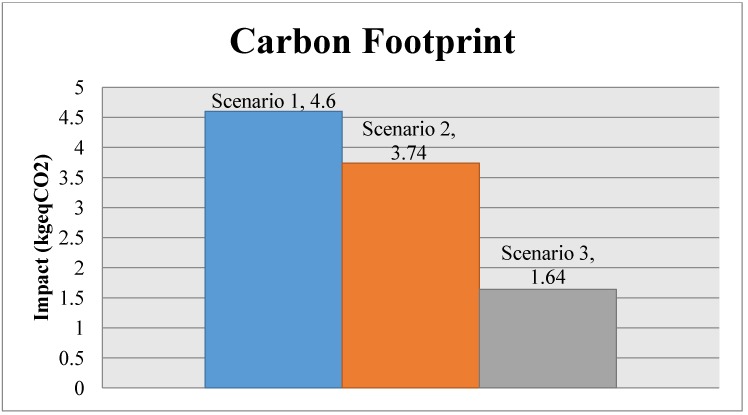
Comparative between scenarios, Carbon footprint.

**Figure 8:**
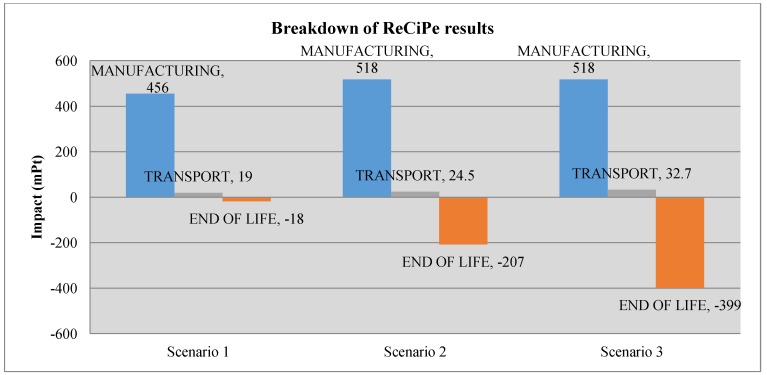
Breakdown of the ReCiPe results.

**Figure 9:**
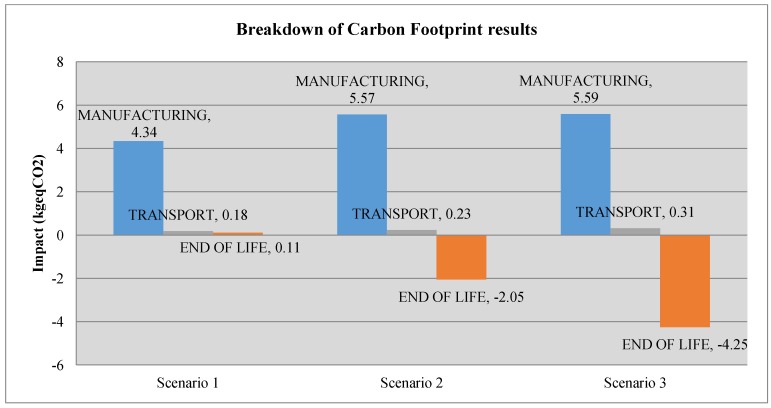
Breakdown of the carbon footprint results.

The following charts (Figure 6 and Figure 7) show the comparison of the environmental impact of the light fitting for the different scenarios, with the ReCiPe results and the carbon footprint. Figure 8 and Figure 9 show, for ReCiPe and carbon footprint results, the breakdown into the different results of manufacturing, end of life and transports.

Looking at the total results chart, we can see that Scenario 3, the one with the whole closed-loop system, is the lowest impact scenario, reducing the environmental impact, while we increase the percentage of light fittings taken back to the factory to be recycled, compared to Scenario 1, where there is a lot of material that is not recycled, according to IEC TR62635.

Looking at Figure 8 and Figure 9, we can see how the choice of the material depending on the EOL (End Of Life) scenario affects the results in the different life cycle stages. For all of the scenarios, the difference of the environmental impact at the transport stage is not relevant. The closed-loop strategy can bolster thinking that it is going to increment the environmental impact due to the increasing of transport distances, as far as the product has to go back to the factory when it reaches its end of life, but we can see that this increase is not relevant, so a closed-loop strategy does not suppose an environmental impact increase due to the increment of transport distances.

It is interesting to see that manufacturing (material + processes) environmental impact increases from Scenario 1 to Scenarios 2 and 3. That is because PC has a greater environmental burden than SMC. According to Scenario 1, SMC would be the better option, because PC is not recycled, as for SMC, so that the total impact is lower for SMC than for PC.

However, with a closed-loop system (Scenarios 2 and 3), where those materials that can be recycled are taken back to the factory to be recycled in the production process, the environmental impact is reduced, as expected, thanks to the end of life impact reduction, due to the recycling.

In Figure 8 and Figure 9 is shown how, while the percentage of recycled light fittings increases, this also increases the reduction of the environmental impact thanks to the recycling process. Finally, from Scenario 1 to Scenario 3, we have a reduction of a 66.7% of mPt and 64.3% of kg_eq_CO_2_.

Scenario 3’s environmental impact could also be reduced by applying design measures. The reduction of weight by means of lower part thickness or reduced light fitting dimensions would reduce the overall weight, decreasing the raw material and transportation impact.

In the next step to improve this research work, a more precise life cycle inventory can be performed. The precision of the datasets could be improved by gathering more data about raw materials and especially recycling processes. The obtention of this information needs supplier collaboration, and a deep analysis of all the stages of the end of life becomes a complex task, where each process, transport, material and recycling, could be analyzed in detail, measuring consumption and analyzing in more detail all the inputs and wastes of each, and thus obtaining more reliable results.

## 5. Conclusions

Material selection has a noticeably relevance to the environmental impact of a product, especially at the end of life. The combination of the material selection with an appropriate business strategy that allows recycling of the material in the factory reduces the environmental impact, making it possible to reduce more than 50% of the impact.

Some business models, like leasing, can realize a noticeable reduction of the environmental impact of a product by means of recycling the old products in the factory to produce new ones, also saving money on raw materials. Furthermore, it can be a good business strategy for this product, whereby you can ensure that the lighting system is going to be replaced in the future with your product, and the company can reduce prices for the customer thanks to the savings of raw material.

This closed-loop strategy is interesting also for society from an ecological point of view. With this business model, materials from products are recycled and reused to produce new products, it not being necessary to consume 100% raw material for every new produced product. Materials used to produce light fittings will have a longer life, by not being downcycled.

As the first analysis, we can see that a closed-loop system is worthy of being studied in detail as a new recycling strategy, as it allows clear environmental impact reductions, so that the improvement of the input information for this model would be helpful to increase the precision of the results.

Furthermore, an economical analysis could be performed to see how this closed-loop affects the profits of the company, as far as the economic factor is a determinant for making decisions. Nowadays, the lighting market is changing thanks to the LED. Before the development of LEDs, industrial light fittings were just a cheap piece of plastic to hold a light tube, and the only maintenance operation was to replace the tube over the years. As far as light fittings were not a technological part, recycling models, like the one proposed, were not interesting. However, nowadays, this product is getting more complicated from a technical point of view, with new materials, new product concepts, where LED luminaires are maintenance free, and new production processes, which is increasing the costs of the product, so that new business strategies could be necessary to face this market change.
